# Anesthetic Management of Children With Propionic Acidemia Undergoing Esophagogastroduodenoscopy

**DOI:** 10.7759/cureus.18168

**Published:** 2021-09-21

**Authors:** Gijo Alex, Edgar E Kiss, Rita Saynhalath, Katie Amy Liu, Sonia D Mehta

**Affiliations:** 1 Anesthesiology and Pain Management, University of Texas Southwestern Medical Center, Dallas, USA; 2 Outcomes Research Consortium, Cleveland, USA; 3 Anesthesiology, University of Florida, Gainesville, USA

**Keywords:** anesthesia, esophagogastroduodenoscopy (egd), propionic acidemia, propofol

## Abstract

Propionic acidemia is a rare genetic disorder of metabolism that predisposes patients to metabolic acidosis, lethargy, neurologic dysfunction, developmental delays, and cardiomyopathy. Perioperative anesthetic management is guided toward mitigating the effects of preoperative fasting times, maintaining normovolemia, and preventing cardiovascular complications secondary to underlying cardiomyopathy. Commonly used anesthetic agents may have undesirable side effects in these patients. Propofol, the lactate in Lactated Ringer's, and neuromuscular blocking agents that undergo ester hydrolysis are poorly metabolized and can lead to metabolic acidosis. Opioids, such as fentanyl and morphine, should be used judiciously in patients with coexisting developmental delays to avoid oversedation and delayed time to resuming oral intake postanesthesia. In addition, inhaled anesthetics have direct myocardial depressive effects and can compromise cardiac function in the setting of pre-existing cardiomyopathy. The perioperative period represents a critical time in this population and appropriate planning is crucial to prevent perioperative morbidity. We present a case of an eight-year-old child undergoing esophagogastroduodenoscopy under general anesthesia and describe the anesthetic concerns we addressed to provide a safe perioperative course.

## Introduction

Propionic acidemia (PA) is a rare autosomal recessive disorder of metabolism and usually manifests shortly after birth with organic acidopathy, poor feeding, lethargy, vomiting, neurologic dysfunction, developmental delays, and cardiomyopathy [1]. Anesthetic management is focused on minimizing the severity of acidosis, maintaining adequate tissue perfusion, and avoiding prolonged fasting times. We describe the anesthetic management and considerations for a child with PA who presented to the operating room for esophagogastroduodenoscopy (EGD).

## Case presentation

An eight-year-old 22-kg child with a past medical history significant for PA, mild developmental delays, generalized hypotonia, dysphagia, and autism presented for EGD with biopsies to evaluate his eosinophilic esophagitis. An electrocardiogram performed prior to the procedure revealed normal sinus rhythm without any evidence of cardiomyopathy. He also had a recent echocardiogram that did not show any evidence of cardiomyopathy. His home treatment plan included a specific diet to control the intake of propiogenic substrates while ensuring normal protein synthesis. Labs revealed normal electrolytes (sodium 140 mmol/L and potassium 4.0 mmol/L) and glucose within a normal range (102 mg/dL).

The patient was preadmitted to the hospital the day before the procedure for medical optimization. He was placed on an infusion of 10% dextrose and normal saline at 1.5 times the maintenance rate and an intravenous formulation of 450 mg of levocarnitine twice daily. The following day, the child was brought to the endoscopy suite after 10 hours of fasting. The dextrose infusion was continued throughout the perioperative period. Standard American Society of Anesthesiologists monitors were applied. Glucose was checked immediately prior to the induction of anesthesia and was a normal value. An inhalation induction was performed incrementally and carefully up to 8% sevoflurane and 100% oxygen. Fentanyl (50 μg) was administered intravenously to facilitate endotracheal intubation. Once endotracheal intubation was completed, the sevoflurane concentration was decreased to 1% to diminish any myocardial depressive effects of the inhaled agent. The EGD was completed in 30 minutes with no complications. The patient was extubated awake and transferred to the postanesthesia care unit. His recovery was uneventful and he was subsequently discharged home on the same day. The patient did not require further follow-up.

## Discussion

**Figure 1:**
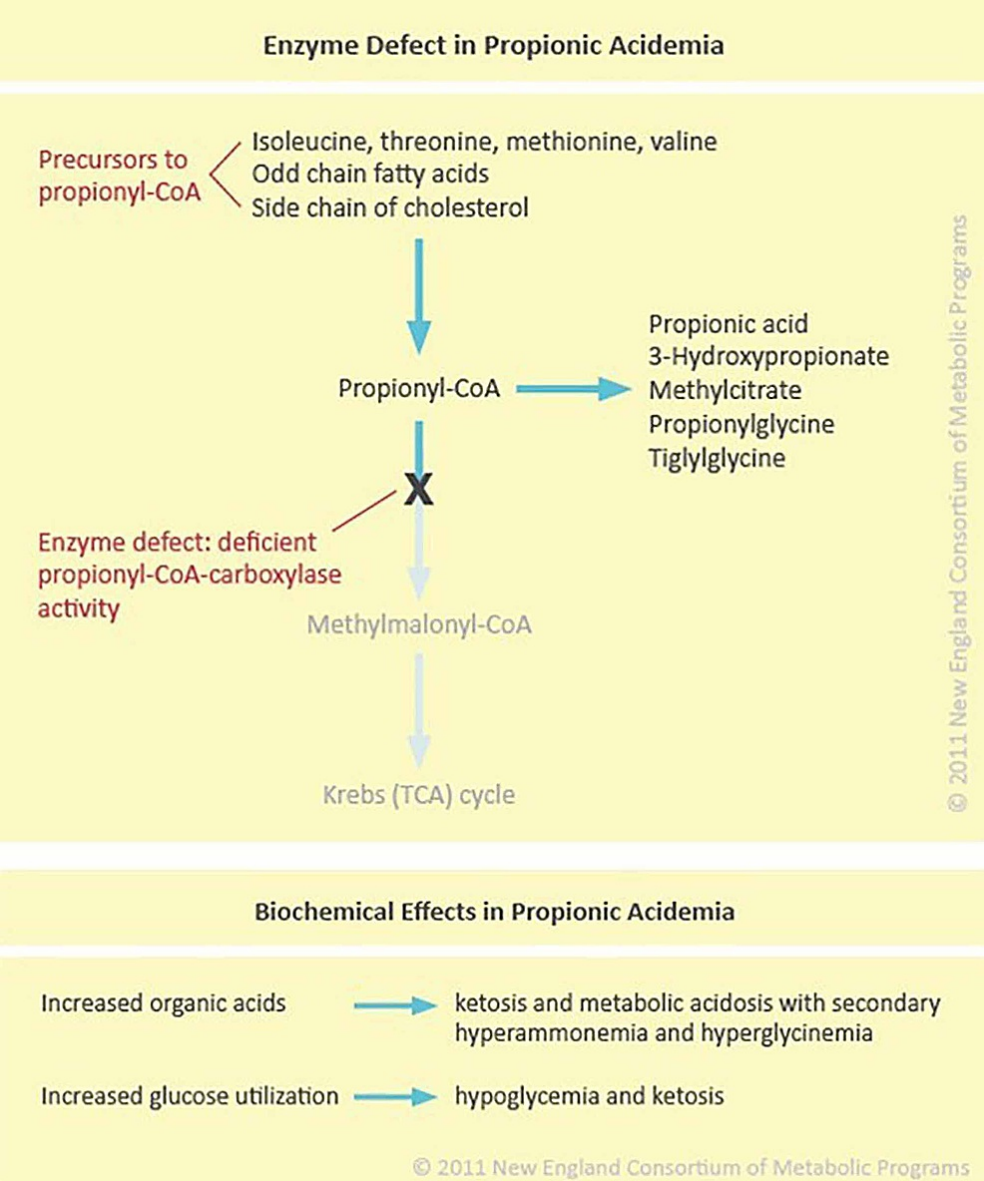
The enzyme defect in propionic acidemia and its effects. CoA, coenzyme A; TCA, tricarboxylic acid. Reprinted with permission from the New England Consortium of Metabolic Programs. Propionic Acidemia. Accessed: September 2, 2020: https://www.newenglandconsortium.org/propionic-acidemia.

PA is an autosomal recessive disorder with a prevalence of 1 in 100,000 live births, but it may be as high as 1:1,000 births in Greenlandic Inuits due to a prevalent mutation [1,2]. Mutations in the propionyl coenzyme A carboxylase alpha (PCCA; chromosome 13q32.3) or propionyl coenzyme A carboxylase beta (PCCB; chromosome 3q22.3) genes lead to a deficiency in the mitochondrial enzyme propionyl coenzyme A carboxylase (Figure 1) [3]. The oxidation of odd-chain fatty acids and certain amino acids, such as methionine, valine, isoleucine, and threonine, leads to the production of propionyl coenzyme A (CoA). Propionyl CoA is then catalyzed into methylmalonyl CoA through propionyl coenzyme A carboxylase. The methylmalonyl CoA is later transformed into succinyl CoA to be used in the Krebs cycle for energy production and biosynthesis. A deficiency in propionyl CoA carboxylase leads to the accumulation of toxic metabolites such as propionic acid, methylmalonic acid, and ammonia. Hyperammonaemia results from the interaction of the substrates of the defective enzymes with other biochemical pathways such as the urea cycle and the tricarboxylic acid (TCA) or Krebs cycle [4].

Propionic acidemia can present in one of the following forms: severe neonatal-onset, intermittent late-onset, or a chronic progressive form. Neonatal-onset PA is characterized by poor feeding, hypotonia, metabolic acidosis, and lethargy in the first few days of life [5]. Up to 30% of afflicted infants may experience seizures. Episodes of metabolic decompensation can result in damage to the basal ganglia and globus pallidus. These metabolic strokes can cause intellectual disabilities of varying degrees and new onset of neurological manifestations: dystonias, tremors, and paraplegia. Intermittent late-onset PA may present after a year or even later. It presents as a metabolic crisis under periods of stress such as surgery, fasting, and illness. In the chronic progressive form, the disease may present as failure to thrive, chronic vomiting, and hypotonia. This chronic form can also lead to other long-term manifestations such as growth restriction, intellectual disabilities, seizures, pancreatitis, long corrected QT (QTc) interval, and severe cardiomyopathy [5].

Primary management of PA includes a low protein diet and administration of levocarnitine. Levocarnitine is important because it conjugates with propionic acid to promote transfer out of the mitochondria and excretion into the urine [1]. In addition, some propose the use of biotin for the management of PA as propionyl CoA carboxylase is a biotin-dependent enzyme and some forms of the disease may respond to biotin therapy [4]. Liver transplantation has also been proposed as a treatment option because it can reduce the number of hospitalizations and improve growth in this patient population [6].

Anesthetic goals for patients with PA aim to prevent acidosis and hypoglycemia to avoid end-organ dysfunction. Preoperative evaluation should include a cardiology consultation to evaluate cardiac function and thorough documentation of baseline neurologic status, including the presence of any developmental delays, hypotonia, or vision impairments due to optic neuropathy [7]. Fasting periods should be minimized. An intravenous dextrose and sodium chloride infusion initiated preoperatively can mitigate hypoglycemia and hypotension.

Propofol emulsions contain polyunsaturated fatty acids, which are metabolized to propionyl CoA and could increase the patient’s level of propionic acid, leading to acidemia [5]. Due to the risk of acidosis, we elected to use sevoflurane as our mainstay anesthetic over a propofol infusion. The lactate in Lactated Ringer's is poorly metabolized by the mitochondria in patients with PA and can also contribute to increased acid load [8]. Opioids can cause oversedation in patients who are developmentally delayed and should be used sparingly to avoid prolonged sedation from exaggerated depressive effects to the central nervous system. Patients with PA can present with cardiac dysfunction. The accumulation of propionyl CoA and other metabolites mediate oxidative damage, compromising the generation of adenosine triphosphate (ATP) via oxidative phosphorylation. The resulting cardiotoxicity can lead to cardiomyopathy and can predispose individuals to fatal arrhythmias. Patients with PA demonstrate QT prolongations associated with ventricular tachycardia and syncope. Medications such as ondansetron and methadone, which can prolong QTc, should be avoided in patients with baseline prolonged intervals or if patients are concurrently taking other medications known to increase QTc. It may be sensible to avoid the use of neuromuscular blocking agents that undergo ester hydrolysis (e.g., succinylcholine, cisatracurium, atracurium, and mivacurium) because the metabolites of these medications include odd-chain organic molecules [9-11]. Also, succinylcholine should be avoided due to the risk of hyperkalemic arrest in myopathic conditions.

For prolonged procedures, anesthetic intraoperative management should include adequate intravenous access to allow for concurrent administration of dextrose-containing fluids and fluid boluses as needed. An arterial line may be placed to measure electrolytes, acid-base status, ammonia, and lactate levels. Postoperatively, patients should be carefully observed for signs of deterioration. Axial hypotonia predisposes patients with PA to a weak cough, atelectasis, and pneumonia, so choosing an anesthetic regimen that avoids prolonged sedation is advisable [5].

## Conclusions

Patients with PA are at an increased risk of perioperative complications. Appropriate preoperative planning to avoid acidosis includes minimal fasting periods and administration of dextrose-containing fluid infusions. Cardiology consultation may be necessary to evaluate for cardiomyopathy and prolonged QTc interval. The commonly used anesthetic propofol is poorly metabolized and can worsen acidosis, especially when used as the mainstay anesthetic. Opioids should be dosed judiciously, with consideration for underlying developmental delays to avoid prolonged sedation and hospitalization. Patients with PA are also at risk for postoperative decompensation and a prolonged observation period in the recovery unit or overnight stay may be prudent.
